# Properties and predicted functions of large genes and proteins of apicomplexan parasites

**DOI:** 10.1093/nargab/lqae032

**Published:** 2024-04-04

**Authors:** Tiffany Fang, Amir Mohseni, Stefano Lonardi, Choukri Ben Mamoun

**Affiliations:** Department of Internal Medicine, Section of Infectious Diseases, Department of Microbial Pathogenesis and Department of Pathology, Yale School of Medicine, New Haven, CT, 06520 USA; Department of Computer Science and Engineering, University of California, Riverside, CA, 92521 USA; Department of Computer Science and Engineering, University of California, Riverside, CA, 92521 USA; Department of Internal Medicine, Section of Infectious Diseases, Department of Microbial Pathogenesis and Department of Pathology, Yale School of Medicine, New Haven, CT, 06520 USA

## Abstract

Evolutionary constraints greatly favor compact genomes that efficiently encode proteins. However, several eukaryotic organisms, including apicomplexan parasites such as *Toxoplasma gondii*, *Plasmodium falciparum* and *Babesia duncani*, the causative agents of toxoplasmosis, malaria and babesiosis, respectively, encode very large proteins, exceeding 20 times their average protein size. Although these large proteins represent <1% of the total protein pool and are generally expressed at low levels, their persistence throughout evolution raises important questions about their functions and possible evolutionary pressures to maintain them. In this study, we examined the trends in gene and protein size, function and expression patterns within seven apicomplexan pathogens. Our analysis revealed that certain large proteins in apicomplexan parasites harbor domains potentially important for functions such as antigenic variation, erythrocyte invasion and immune evasion. However, these domains are not limited to or strictly conserved within large proteins. While some of these proteins are predicted to engage in conventional metabolic pathways within these parasites, others fulfill specialized functions for pathogen–host interactions, nutrient acquisition and overall survival.

## Graphical Abstract

**Graphical Abstract:**
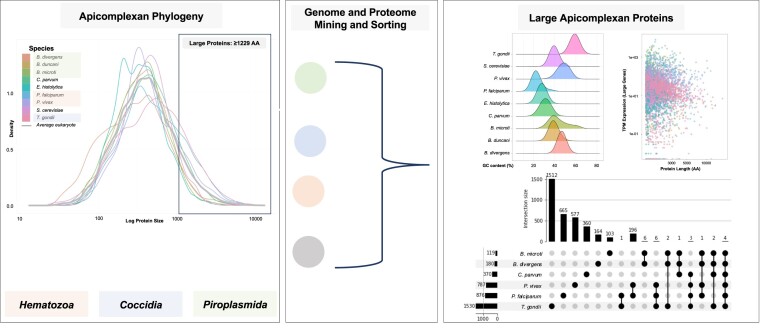

## Introduction

The phylum Apicomplexa encompasses many species, several of which are responsible for significant diseases affecting humans, livestock and companion animals. Among these vector-borne pathogens are members of the genera *Plasmodium* and *Babesia*, which are responsible for human malaria and babesiosis, respectively. The mosquito-transmitted *Plasmodium falciparum* alone accounts for over 99.7% of fatal human malaria cases with 0.5 million deaths every year (1,2). On the other hand, tick-transmitted *Babesia* species can induce acute to life-threatening infection in humans and animals (3,4), with hospitalized babesiosis patients exhibiting severe symptoms at a rate exceeding 58% and a 1.6% death rate (5). Human babesiosis is attributed to several species of *Babesia*, with *Babesia microti* responsible for the majority of clinical cases reported so far (6). Other species include *B. divergens*, *B*. MO1, *B. duncani* and *B. venatorum* (4). The clinical presentation of human babesiosis varies widely, ranging from flu-like fever to severe complications such as anemia, disseminated intravascular coagulation, acute respiratory distress syndrome or even fatal outcomes. In Europe, *B. divergens*, a natural cattle pathogen, is the primary agent responsible for human babesiosis (3). Other members of the Apicomplexa phylum include *Cryptosporidium parvum*, a human and zoonotic pathogen transmissible through fecal matter that causes acute gastroenteritis (7). *Toxoplasma gondii*, the etiological agent of toxoplasmosis, affects ∼30% of the global human population and poses the risk of severe complications such as encephalitis, myocarditis and pneumonitis when transmitted through raw meat or contaminated water (8). As obligate intracellular parasites capable of salvaging nutrients from their mammalian host, apicomplexans do not need to support complex metabolic and biochemical processes; as a result, they generally have smaller genomes than free-living eukaryotes, ranging from around 8 to 130 Mb (9).

A recent study by Singh *et al.* shed light on the genome of *B. duncani*, revealing a compact 7.55 Mb genome housing 4222 genes with an average length of 1656 bp (9). Approximately 60% of this genome is involved in protein coding. *Babesia duncani* shares 842 core proteins (comprising 20% of its proteome) with other apicomplexan parasites; out of *B. duncani*’s 1242 unique proteins, around 70% lack functional annotations (9). Among these hypothetical proteins, BdWA1_000001 stands out as a polypeptide consisting of 11 561 amino acids. In a genome characterized by an average protein length of ∼500 amino acids, the presence of genes encoding such large proteins raises fundamental questions concerning their significance in parasite biology, development and virulence.

The prevalence of such large proteins extends beyond *B. duncani*, with other apicomplexan species boasting even larger proteins. For instance, the *T. gondii* proteome encodes seven proteins exceeding 10 000 amino acids, with its largest protein comprising 17 226 amino acids (10,11). Similarly, *C. parvum*, *P. falciparum* and *Plasmodium vivax* all encode proteins exceeding 10 000 amino acids (10,11).

We conducted a comprehensive bioinformatics analysis encompassing the genomes and proteomes of seven apicomplexan parasites, alongside two reference groups: one parasitic protist *Entamoeba histolytica* and the extensively studied nonparasitic unicellular organism *Saccharomyces cerevisiae*. Our aim was to uncover discernible patterns in the composition and functionality of large proteins within these organisms. Our analysis of the apicomplexan genomes revealed that the distributions of protein sizes skewed toward the right, revealing a tendency for larger genomes to accommodate larger proteins. Moreover, these larger proteins exhibited lower levels of expression, and their amino acid usage profiles closely mirrored those of their respective genomes. From a functional perspective, we observed that Gene Ontology (GO) functional annotations were not evenly spread across protein sizes. Several functional categories, such as those related to transport, metabolism and nucleic acid regulation, were significantly overrepresented in larger proteins across all seven species. Within these large proteins, we identified conserved domains with similar functions, as well as species-specific domains crucial for various aspects of parasite life cycles. These specialized domains facilitated processes such as host cell invasion, immune evasion and sustained proliferation. Importantly, although these domains were present in different species, they were not restricted to large proteins, suggesting their adaptable roles in diverse biological contexts.

## Materials and methods

### Genomic data acquisition

Genome assemblies were obtained from Singh *et al.* (9) (*B. duncani*) and VEuPathDB (*B. divergens*, *B. microti*, *C. parvum*, *P. falciparum*, *P. vivax*, *T. gondii*, *E. histolytica* and *S. cerevisiae*), using the reference genome for each species. The primary data collected included protein length, coding sequence (CDS) length, transcription levels (transcripts per million, TPM), number of transmembrane domains and computed GO function IDs. Protein and transcript sequences were also collected. Codon usage data for whole genomes were taken from organism-specific databases such as AmoebaDB, CryptoDB, FungiDB, PiroplasmaDB, PlasmoDB and ToxoDB.

### Figure generation

Heatmaps were generated in R using the ‘heatmap2’ function within the ‘gplots’ package, size histograms of Figure 2 were generated with fixed bins in Excel and all other plots were created with the ‘ggplot’ package.

### Protein size thresholding

Using summative data from 485 eukaryotic proteomes (12), the average eukaryotic proteome size and protein length were computed to be 17 215 proteins and 344 amino acids, respectively. Operating under the statistical assumption that eukaryotic protein sizes tend to approximate a log-normal distribution (13), a set of 17 215 normally distributed ‘log-protein size’ random values was generated around a mean of ∼2.53 with a standard deviation of ∼0.33 (calculated using quartile data on the proteomes). Based on this statistical analysis, a *large protein* is a protein whose length exceeds the 95th percentile of the log-normal distribution, namely 1229 amino acids.

### Analysis of genomic properties

The normalized large protein quotient was calculated by dividing the number of large proteins in a specific genome by the genome size in Mb. This value for the ‘average eukaryotic genome’ was calculated by averaging the genome sizes of the previous 485 eukaryotes and dividing them by the average number of large proteins. Since ‘large proteins’ are determined to be the 95th percentile, they would account for 5% of the whole average eukaryotic proteome, thus being 860 proteins. The guanine and cytosine (GC) content was calculated using the ‘GC’ function in the BioPython package of Python.

### Analysis of protein properties

Codon frequency data are reported in frequency per thousand; genome-wide amino acid usage was calculated by adding the frequencies of all codons that encode each amino acid. The amino acid frequencies were calculated as the fraction of occurrence across the entire protein.

### Statistical GO analyses

A custom script was used to extract the GO annotations of all large proteins; for each annotation, the number of occurrences within the large proteome and that within the whole proteome were recorded (Figure 5A). A *P*-value was calculated for observing a greater number of occurrences of this annotation according to a hypergeometric distribution, with the threshold of significance set to 0.001; this identified 53 GO classes to be significantly overrepresented.

### Motif prediction

Conserved domains for the largest proteins in Supplementary Table S1 were determined using CD-Search (14) based on amino acid sequence. Conserved domains for large proteins in general in Figure 6 were determined using the get_cdd() function within the ‘ragp’ R package (14). PFam domains were identified using OrthoMCL (15). We utilized DIAMOND (16) and configured it with the ‘--sensitive’ option to cluster and identify homologous gene hits of at least 40% identity and 80% coverage across all species mentioned in this study. Additionally, we visualized all overlapping orthologous gene sets using the ‘UpSetPlot’ Python package shown in Figure 7A and B.

## Results

### Size distribution of apicomplexan proteins

**Figure 1.**
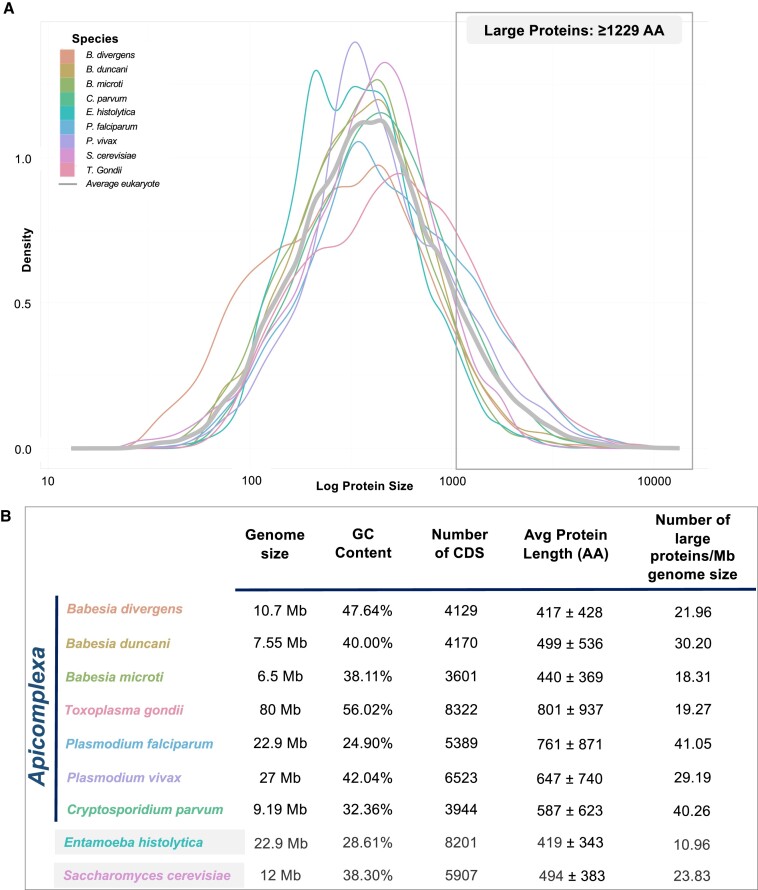
(**A**) Probability density function estimation of log size distribution of proteins in all genomes, smoothed with Gaussian kernel and compared with the ‘average eukaryotic protein size distribution’. (**B**) Genomic overview including genome size, GC content, number of CDSs, average protein length and number of large proteins normalized over genome size in Mb.

**Figure 2.**
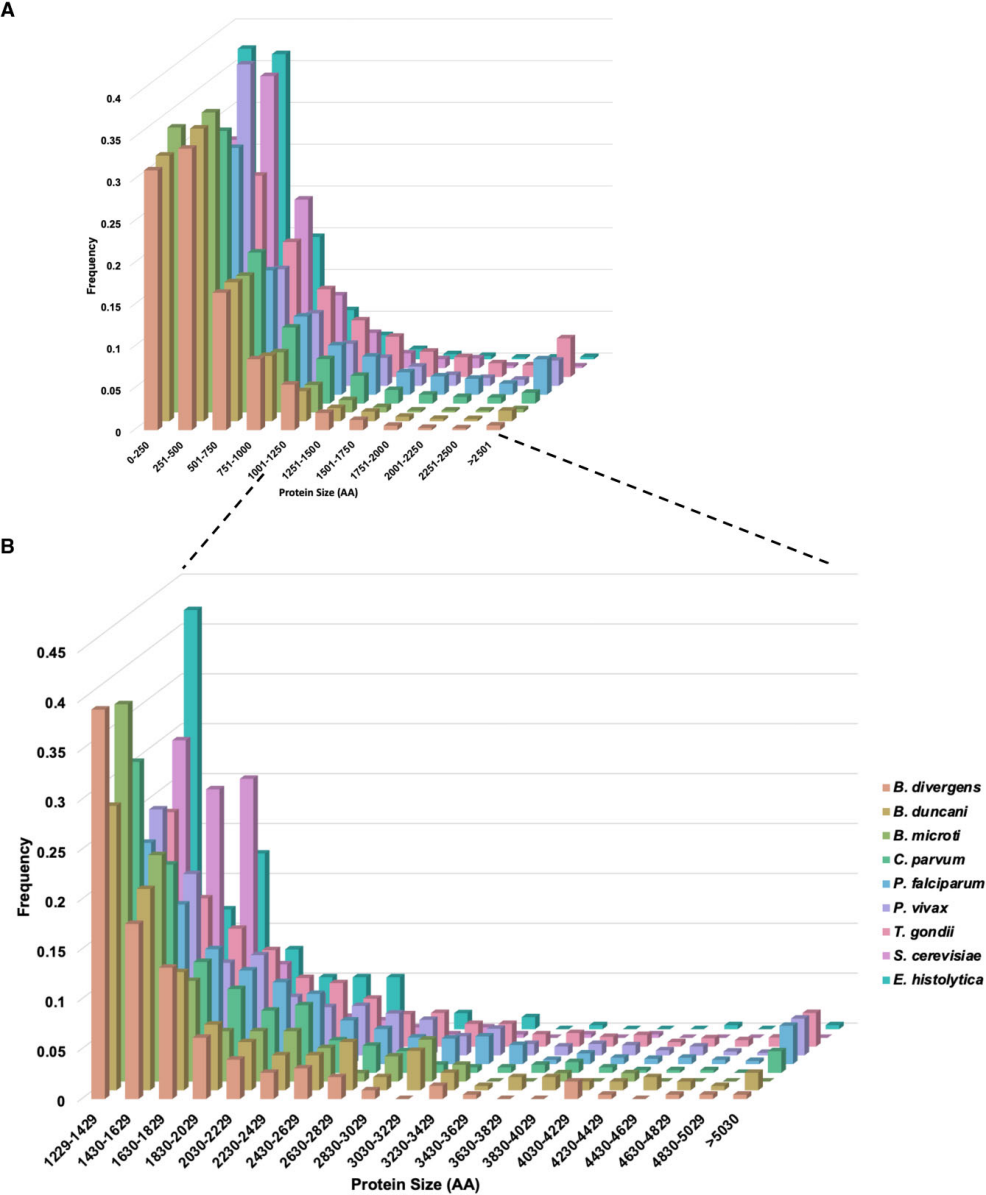
**(A**) Histogram showing distribution of protein size relative to number of total proteins in all parasite proteomes divided into size bins of 250 amino acids. (**B**) Distribution of protein size ratio over large proteins in all parasites.

Despite the fact that all the species analyzed in this study belong to the Apicomplexa phylum, their genomes exhibit significant diversity. These parasites possess genomes of varying sizes, ranging from 6.5 Mb in *B. microti* to 80 Mb in *T. gondii* (Figure 1B). We calculated a normalized number of large proteins per species (Figure 1B), with a higher value representing a greater number of large proteins per Mb of genome size and more investment of the species into maintaining large proteins. All species in this study show greater large protein density than the average eukaryotic genome (5.157 large proteins per Mb); large protein density is also higher for Apicomplexa than the two outgroups. Notably, all genomes in this study display a right-skewed distribution, with a preponderance of smaller proteins in each size category (Figure 2). However, when we analyze protein size distribution on a logarithmic scale, it conforms to a Gaussian distribution (Figure 1A), a pattern consistent with the average eukaryotic genome. Using this statistical approach, we defined ‘large proteins’ in this study as those exceeding the 95th percentile of the distribution, corresponding to proteins longer than 1229 amino acids. This encompasses 235 proteins in *B. divergens*, 228 in *B. duncani*, 119 in *B. microti*, 1542 in *T. gondii*, 940 in *P. falciparum*, 788 in *P. vivax*, 370 in *C. parvum*, 251 in *E. histolytica* and 286 in *S. cerevisiae*.

### GC content and protein size

**Figure 3.**
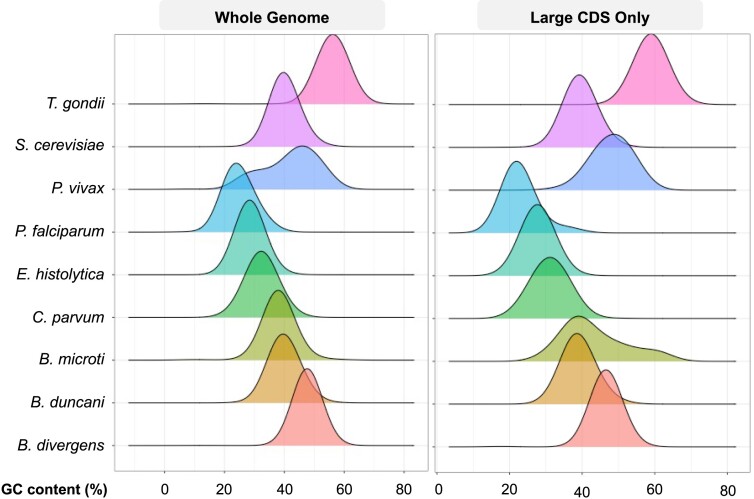
GC content distribution by CDS length for whole genome and large genes of apicomplexan and outgroup species studied.

By examining the GC content of CDSs, we found that GC content distribution varies among species (Figure 3), with *P. falciparum* displaying the lowest average GC content at 24.9% and *T. gondii* the highest at 56.02% (Figure 1B). Interestingly, the GC content distributions in long CDSs (encoding large proteins) often mirror the overall GC content of the species. However, for certain species, large CDSs exhibit either more extreme (e.g. *P. falciparum*’s large CDS has an even lower average than the overall) or different (e.g. *P. vivax* and *B. microti*) GC content distributions compared to the overall genome. While most GC distributions approximate a normal distribution, the large CDSs of *B. microti* and whole genome of *P. vivax* appear bimodal. Moreover, GC content does not appear to be strongly correlated with CDS length, with Pearson correlation coefficient below 0.35.

### Expression levels of genes encoding large proteins

**Figure 4.**
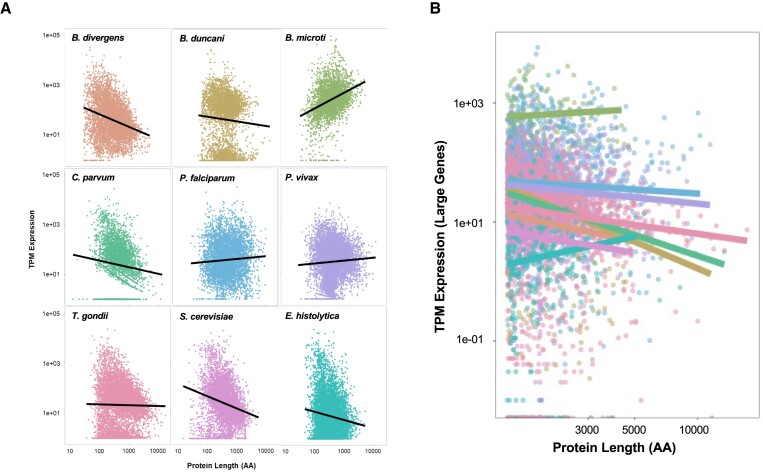
Log_10_-transformed scatterplots of TPM expression level by protein length across (**A**) the whole genome and (**B**) large proteins of each species, with trendlines for each.

We further examined the expression profile of genes encoding from all selected species using transcriptional data available in VEupathDB. We found that larger genes encoding large proteins tend to be expressed at lower levels compared to shorter genes, which display a more diverse expression profile (Figure 4A). This trend persists across various species. Further investigation of the expression levels of the largest genes (Figure 4B) showed a similar trend with shorter genes having a more varied range of possible expressions, whereas large genes were almost exclusively expressed at low levels.

### Amino acid usage in large proteins

When examining amino acid usage in large proteins, we observed significant variation depending on the species and amino acid type (Supplementary Figures S1 and S2). In the case of *B. duncani*, lysine fraction held a general negative correlation with length across all proteins, but an opposite positive correlation when focusing on large proteins. In contrast, the arginine fraction displayed a general negative correlation with length across all *B. duncani* proteins including large proteins, whereas the cysteine fraction displayed a general positive correlation with length across all proteins including large proteins (Supplementary Figures S1 and S2). In the case of *P. falciparum*, asparagine fraction displayed a strong positive correlation among average-size proteins but was less correlated to length in large proteins (Supplementary Figure S4). In contrast, the arginine fraction displayed a general negative correlation with length across all *P. falciparum* proteins excluding large proteins (Supplementary Figures S2–S4). These patterns of amino acid usage align with codon usage patterns (Supplementary Figure S3) as well as GC fraction. For example, the high AT content of *P. falciparum* supports the prevalence of the AAU codon and consequently the abundance of asparagine in its proteins.

### GO functional analyses of large proteins across Apicomplexa

**Figure 5.**
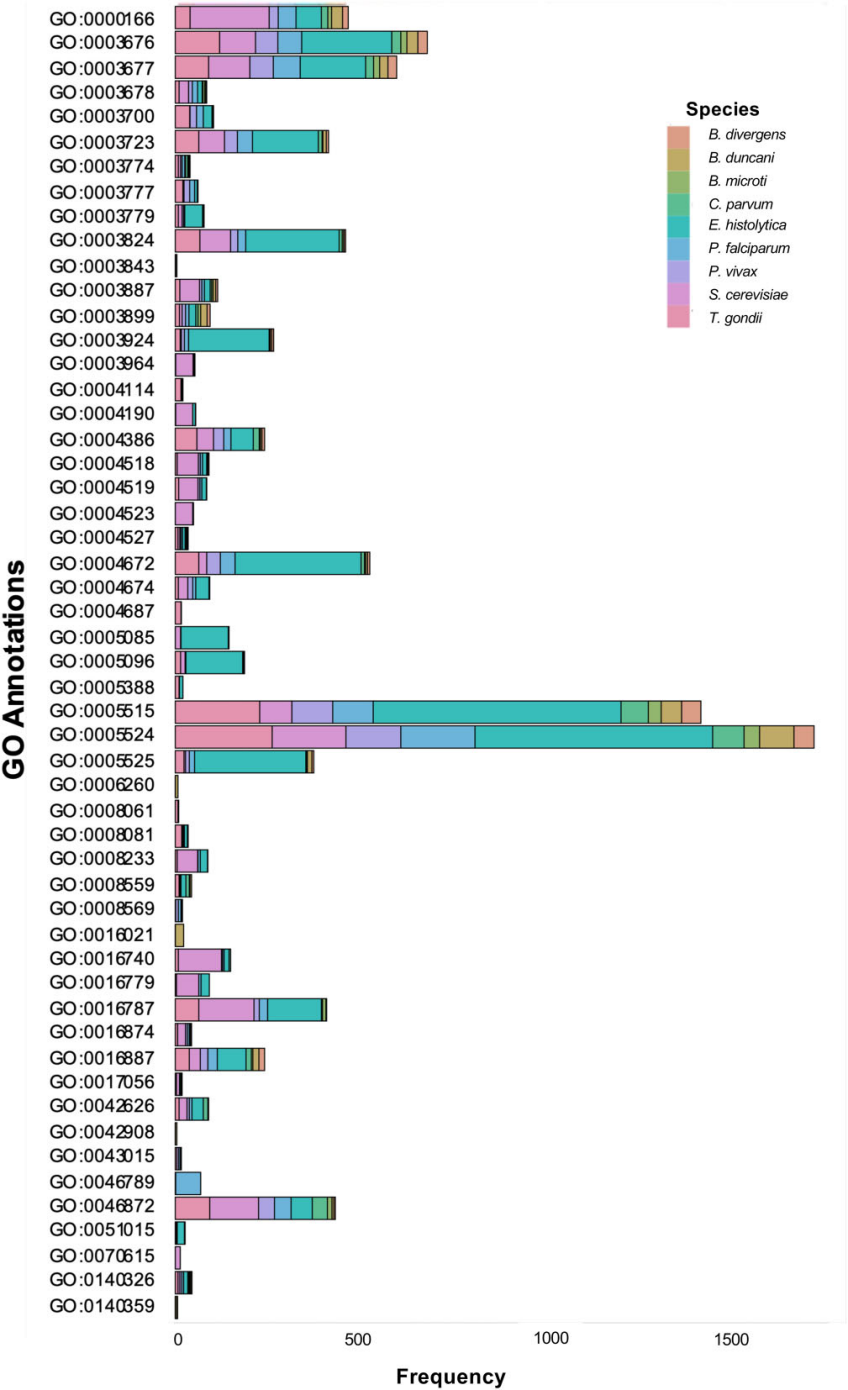
Frequency of occurrence of significantly overrepresented (*P* < 0.001) GO classes in large proteins of all parasitic genomes and outgroups determined via hypergeometric distributions (GO annotation glossary is provided in Supplementary Table S3).

To discern the role of large proteins, we investigated whether the predicted functions of large proteins differ from those of average-size proteins. Assuming a hypergeometric distribution, we identified significantly enriched categories within each large proteome, with a *P*-value <0.001, except for *B. divergens*. Of all the GO functional classes attributed to large proteins, 53 unique GO functions were found to be overrepresented (Figure 5). Common highly represented annotations from all species examined include functions related to protein binding (GO:0005515), ATP binding (GO:0005524), DNA binding (GO:0003677) and nucleic acid binding (GO:0003676). In addition to the GO annotations generally overrepresented in all large proteomes, five parasites exhibited distinctive and disproportionately overrepresented annotations in their large proteomes. Notably, *B. duncani*’s large proteins showed an overrepresentation of functions related to nuclear export and aminoacylation, *Babesia microti*’s large proteins were characterized by a higher representation of functions associated with protein ubiquitination, whereas *P. falciparum*’s large proteins displayed unique and parasite-specific annotations, with a highly significant presence of host cell binding functions (*P* = 3.21e−39) compared to the rest of the proteome. In the case of *P. vivax*, unique functions enriched in its large proteins included cysteine peptidases and gamma-tubulin binding proteins, suggesting potential involvement in processes such as protein degradation, amino acid utilization, protein secretion, cytoskeletal organization (17) or nucleation of microtubule heterodimers (18)*. Toxoplasma gondii*’s large proteome exhibited an overrepresentation of phosphodiesterase, chitin binding and zinc binding annotations. Notably, the chitin-binding-like domains of *Toxoplasma* possess lectin properties that play a pivotal role in host cell binding (19). While zinc ion binding was specifically enriched in *T. gondii*’s large proteins, the ‘metal ion binding’ annotation was shared among large proteins across the Apicomplexa, indicating a potential role of large proteins in zinc salvage pathways (20). Furthermore, domains linked to functions such as protein phosphorylation, transcription and xenobiotic transport were overrepresented in large proteins from parasites but not in the model organism *S. cerevisiae*. *Entamoeba histolytica* displayed significant representation across most of these GO categories, indicating a commonality among Apicomplexa. Altogether, the functions likely served by large proteins can be categorized into three main groups: transport and signaling, metabolic processes, and nucleic acid or protein synthesis.

### Comparative analysis of large protein orthologs in apicomplexan parasites and outgroup organisms

**Figure 6.**
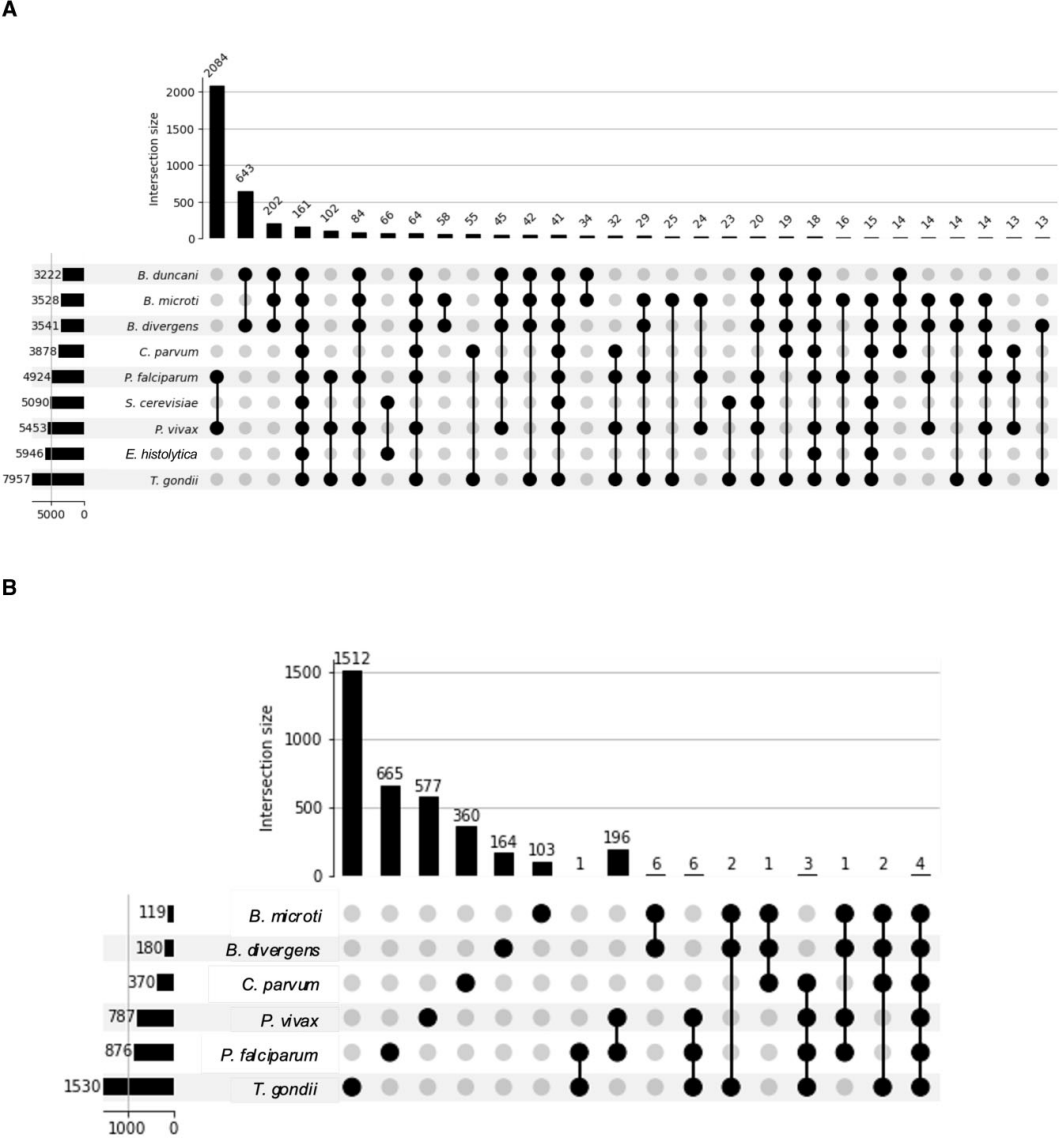
UpSet plots illustrating (**A**) the top 30 ortholog groupings of species in this study (top: total number of shared proteins among different organisms; bottom left: number of shared genes per organism) and (**B**) ortholog intersections in large proteins.

To investigate the potential functional similarities among large proteins within Apicomplexa, we utilized the DIAMOND software package to identify shared ortholog groups between the complete proteomes and the subsets of large proteins from seven apicomplexan parasites and two outgroup species (Figures 6A and B, respectively). Among the 30 species groupings with the greatest number of orthologs, *P. falciparum* and *P. vivax* exhibit a shared set of 2084 gene orthologs, while *B. duncani* and *B. divergens* share 643 orthologs. Collectively, all species in the study share 161 orthologs across their entire proteomes. Intriguingly, apicomplexan parasites share a greater number of orthologs with *S. cerevisiae* (41 orthologs) than with *E. histolytica* (18 orthologs). However, these orthologs are not predominantly large proteins. Specifically, among the large proteins, only 196 orthologs are shared between *P. falciparum* and *P. vivax*, with all other intersections containing <10 orthologs (Figure 6B). Not all species are depicted due to the absence of orthologs in some cases, highlighting the lack of orthologs among all the large proteomes included in this study.

### Functional domain conservation among largest proteins across diverse species

To investigate the functional conservation of the largest proteins among each of the large protein sets across different apicomplexan species, various motif analyses were conducted, including predictions for transmembrane domains, glycosylphosphatidylinositol (GPI) anchors and conserved motifs (Supplementary Table S1). The search for transmembrane domains revealed notable differences among species. For instance, the largest protein in *B. duncani*, BdWA1_000001, has 18 potential transmembrane domains, whereas the largest protein in *P. falciparum*, PF3D7_0628100, has only 2 potential transmembrane domains. No transmembrane domains were identified in the largest proteins in the other species analyzed in this study. Conversely, GPI anchor prediction tools did not yield any hits for any of the largest proteins. Subsequent conserved-domain searches were conducted on the largest proteins from each species, and the identified domains were further classified into functional groups such as transport and signaling, canonical metabolic processes, and nucleic acid or protein synthesis (Supplementary Table S1). The only ‘largest protein’ that returned no results is the *B. duncani* BdWA1_000001, so an additional conserved-domain search was made on the closest size analog of BdWA1_000001 to possibly predict its function. Unique conserved-domain hits are summarized in Supplementary Figure S1, while ortholog groups and PFam domains can be found in Supplementary Table S2. Overall, our analysis revealed a degree of functional similarity among the conserved domains in different species. Notably, the largest protein in *B. divergens* contains a RAB escort domain linked to membrane trafficking (21) and a chorein-like domain involved in intracellular protein transport and lipid transfer (22). In *B. microti*, the largest protein harbors a vWF (von Willebrand factor) domain found in plasma proteins such as complement factors, integrins, collagens and other matrix proteins involved in multistep signal transduction pathways (23). Further analysis using PFam domain search found six domains within this protein commonly found in proteins of the AAA (ATPases associated with various cellular activities) family. More specifically, these domains belong to the dynein-related subfamily, suggesting a potential involvement in transport functions (24). In *C. parvum*, the largest protein displayed 29 conserved domains linked to metabolic processes, including polyketide synthesis and fatty acid metabolism (25,26). One of the domains found in the largest protein of *C. parvum* is a Kringle domain found in proteins involved in blood clotting (27). The largest protein in *P. falciparum* contains a HECT domain involved in ubiquitination, and three copies of ankyrin repeats associated with various functions such as transcriptional regulation, ion transporter and signal transduction (28). Similarly, *T. gondii*’s largest protein also contains a HECT domain. The most notable domain found in the largest protein of *P. vivax* is associated with pseudouridylate synthase function, which is important for the modification of uracil bases to pseudouridine (29). One region was also similar to TRAFs, which are proteins that regulate cell survival and stress responses in the immune system. Other domain hits were more parasite-specific, including a malarial adhesin-like domain and a microneme/rhoptry antigen.

**Figure 7.**
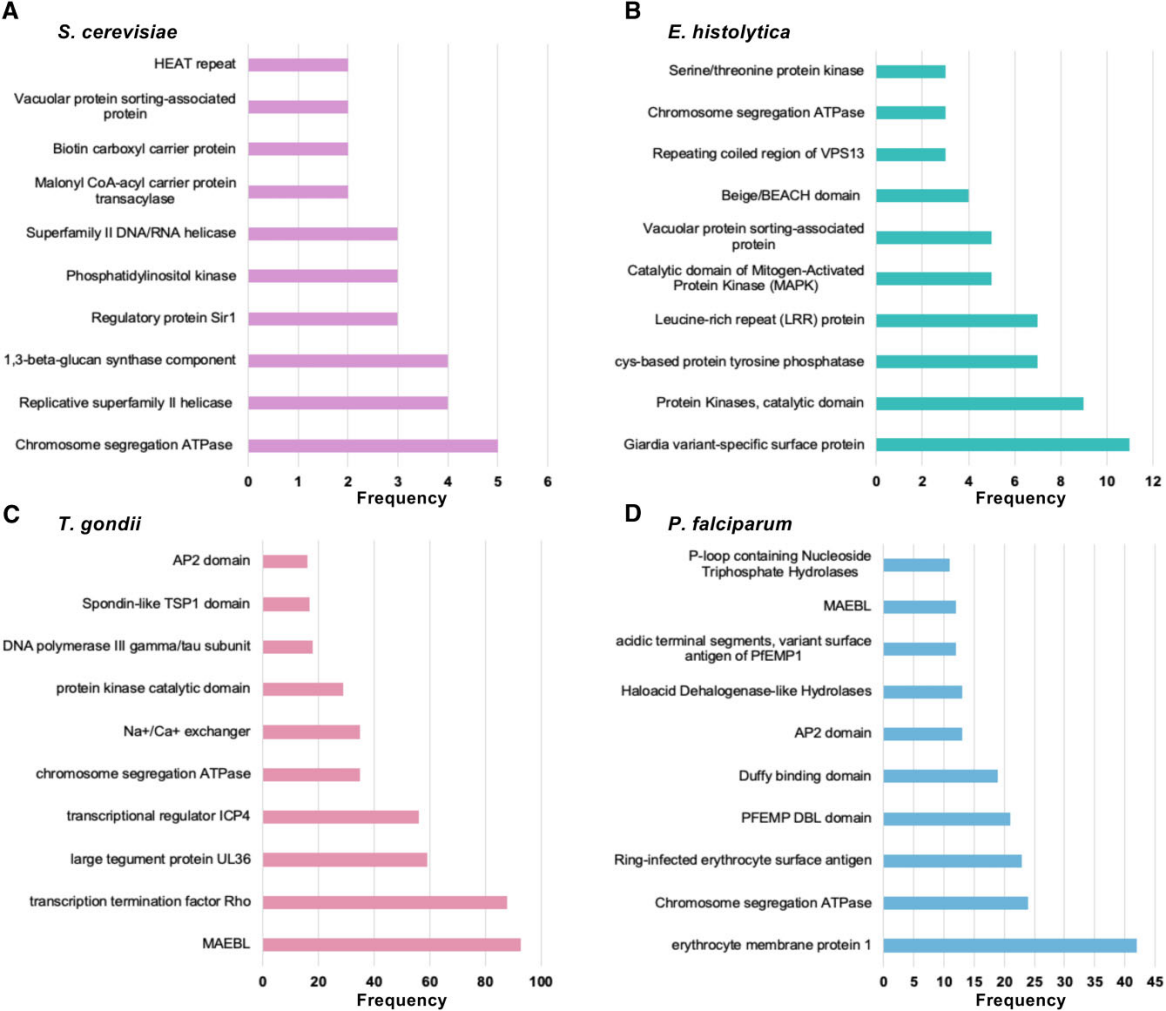
Top 10 most common conserved domains among large proteins of controls (**A**) *S. cerevisiae* and (**B**) *E. histolytica* as well as select Apicomplexa (**C**) *T. gondii* and (**D**) *P. falciparum*.

To broaden our understanding, conserved-domain searches were extended to outgroup species and select Apicomplexa species. Outgroups displayed fewer conserved-domain hits than the apicomplexan species analyzed. In general, these proteins were found to encode housekeeping functions. For example, some of the most common domains in *S. cerevisiae* large proteins are involved in mitosis (chromosome segregation and replicative helicases), cell wall synthesis, lipid synthesis and intracellular trafficking (Figure 7A). Most of the common conserved domain hits in *E. histolytica* large proteins are associated with similar functions, notably transport, cell cycle control and signal transduction. The largest protein of *E. histolytica* contains a *Giardia* variant-specific surface protein (VSP) domain (Figure 7B) found in cysteine-rich VSP proteins, involved in antigenic variation and survival of the parasite in the host (30). In *T. gondii*, common conserved domains include those found in merozoite adhesive erythrocytic binding proteins (MAEBL), herpes tegument protein UL36 (31), regulatory protein ICP4 (32) and AP2 factors (Figure 7C). Five of *P. falciparum*’s 10 most common hits include various domains of the *P. falciparum* erythrocyte membrane protein 1 (PfEMP1), which is expressed by malaria parasites during their blood-stage development and serves as both surface antigen and adhesion molecule with a critical role in virulence (33) (Figure 7D). This is consistent with data from VEuPathDB annotations, indicating that 61 of the 940 large proteins of *P. falciparum* are classified as PfEMP1 proteins. Similarly, of the 235 large proteins of *B. divergens*, 36 are annotated as members of the variable erythrocyte surface antigen.

## Discussion

This study is the first of its nature to investigate the properties, distribution, expression and predicted functions of large proteins of apicomplexan parasites. These proteins have been historically understudied, with their functions remaining poorly elucidated even in model organisms. This limited knowledge stems from the inherent challenges associated with expressing these proteins as full-length polypeptides to assess their biochemical, cell biological or structural properties. Moreover, while some pathogens, such as *T. gondii*, benefit from well-established genetic tools facilitating gene-specific and large-scale functional analyses through gene deletion, editing or regulation, genetic manipulation remains challenging for most apicomplexan parasites. In some cases, such as *B. microti* or *B. duncani*, this field of research is in its infancy (34–37). The use of bioinformatics tools offers a unique opportunity to start exploring the hidden world of large proteins in the biology and pathogenesis of these parasites. Our investigation revealed that very large proteins occur infrequently and are also not highly expressed. Additionally, we found a higher abundance of large proteins, including those considered ‘very large’, within the Apicomplexa group compared to the nonparasitic unicellular outgroup *S. cerevisiae* and the parasitic protist outgroup *E. histolytica*.

The two populations with bimodal GC distributions show interesting trends. For *B. microti*, there does not seem to be a clear functional difference between the pool of genes with a median GC of 38% and the one of 60%; the bimodality could be due to the fact that there are only 53 genes encoding large proteins in *B. microti*, so a larger sample size may yield a greater degree of normality. For *P. vivax*, across both the 30% GC median group and the ∼45% GC median group, predominant functional annotations include hypothetical conserved *Plasmodium* genes, *Plasmodium* interspersed repeat (PIR) genes and zinc finger genes. PIR genes comprise one of the largest multigene families in *Plasmodium*, with their expression indicative of blood-stage parasitic infection but not necessarily playing a role in antigenic variation (38). The zinc finger protein is a common DNA-binding domain in several transcription factors, not necessarily indicative of differential expressions of any function in particular.

Based on trends in abundance of amino acids, it may be possible to make inferences on the predicted structures of large proteins. Since most higher level computational forms of structure prediction (e.g. AlphaFold) are unavailable to large proteins, any insight into potential structures or domains present in these proteins would provide information into their functions and reasons for evolutionary conservation. However, the role of cysteine abundance patterns remains unclear.

Through GO annotation, we identified specific functional classes that large proteins are more likely to belong to. These functional classes primarily fall into three categories: transport and signaling, metabolic processes, and nucleic acid or protein synthesis, annotations more likely to be essential at least in the blood stages of *Plasmodium* parasite. Within the category of large proteins, smaller proteins ranging from ∼1000 to 3000 amino acids tend to be better annotated than those exceeding 10 000 amino acids. This discrepancy emphasizes the limitations of predicting the functions of less annotated very large proteins based solely on the overrepresentation of a particular functional class.

Based on conserved domain hits, it is evident that most large proteins fulfill essential housekeeping functions, with specific roles varying among different species. These roles commonly include the maintenance of cell cycle function, transport processes and metabolic functions. Additionally, each species exhibits its own set of species-specific domains. For example, *S. cerevisiae* expresses domains related to fungal cell wall synthesis, *E. histolytica* expresses *Giardia* variant-specific surface domains and *P. falciparum* expresses PfEMP1-like domains. Interestingly, *E. histolytica* likely shares domains similar to *Giardia* due to their common status as intestinal protozoan parasites (39). However, the origin and specific functions of these proteins in *E. histolytica* remain to be determined. These variant surface domains play a crucial role in antigenic variation among parasites, contributing to their infectivity. Similarly, PfEMP1, a multigene family of highly polymorphic erythrocyte surface proteins, aids in immune evasion and erythrocyte sequestration to promote intraerythrocytic growth and sustain chronic infections (33). Our analysis showed that several large proteins of *T. gondii* and *P. falciparum* contain MAEBL and AP2 domains. MAEBL, originally found in *P. gallinaceum*, mediates binding of parasites to the erythrocyte and is highly conserved among *Plasmodium* species (40). AP2 genes encode DNA-binding proteins serving as transcription factors, with the AP2 domain being highly conserved among Apicomplexa, including *Babesia* and *Theileria* species (41). These domains, however, were found in the large proteins of *Babesia* species, but appear to occur primarily in proteins shorter than 1229 amino acids (9,41).

While our knowledge about BdWA1_000001, the 11 561-amino-acid-long protein of *B. duncani*, remains very limited, conserved-domain searches of the largest proteins in other apicomplexan proteomes may provide insight into their functions. Some of these domains are species-specific, such as malarial adhesin-like domains of *P. vivax*, which could play a role in parasite attachment and host cell entry (42). However, even the domains that are not specific to apicomplexans could possibly play very specialized roles in parasite development and interaction with the host. The largest protein in *C. parvum* returned 29 domain hits, most of which belonged to polyketide synthesis domains, NAD binding domains and phosphopantethine prosthetic groups, which together appear to be involved in carbon chain elongation for fatty acid synthesis (43). This pathway is likely maintained as *C. parvum* salvages free fatty acids and uses its own elongation mechanisms to generate complex lipid structures (44). *Babesia divergens* and *B. microti* both have more transport-related domains, with *B. divergens*’s domains being involved in intracellular transmembrane protein trafficking and Rab-mediated vesicle transport and *B. microti*’s domains more pertaining to nuclear functions such as transcription and export of ribosomes (45–47). The Rab-based vesicle transport mechanism is crucial to the efficacy of intracellular parasite transport, as it modulates both endocytic uptake and secretion to micronemes and rhoptries (48). Although the conserved domains of these largest proteins vary by genome, they all serve essential functions, particularly in transport and signaling or metabolism.

While conserved domains are vital for the functions of large proteins across species, it is crucial to recognize that these domains are not exclusively present in large proteins. Current data do not indicate the presence of selective pressures promoting highly conserved large proteins essential to Apicomplexa functionality. Large proteins across species tend to share functional annotations related to general macromolecule synthesis, particularly fatty acid synthesis and transport mechanisms. These proteins serve as intracellular modulators of secretion pathways, as well as catalysts for replication and synthesis. Further research, encompassing cell biological and genetic studies, is imperative to unravel the functions of these large proteins and their significance in the pathogenesis and virulence of protozoan parasites.

## Supplementary Material

lqae032_Supplemental_Files

## Data Availability

The data underlying this article are available in the article and in its online supplementary material.

## References

[1] Maier A.G., Matuschewski K., Zhang M., Rug M. Plasmodium falciparum. Trends Parasitol. 2019; 35:481–482. 30595467 10.1016/j.pt.2018.11.010

[2] World Health Organization World Malaria Report 2022. World Health Organization. 2022; 14–16.

[3] Ord R.L., Lobo C.A. Human babesiosis: pathogens, prevalence, diagnosis and treatment. Curr. Clin. Microbiol. Rep. 2015; 2:173–181. 26594611 10.1007/s40588-015-0025-z PMC4649939

[4] Renard I., Ben Mamoun C. Treatment of human babesiosis: then and now. Pathogens. 2021; 10:1120. 34578153 10.3390/pathogens10091120 PMC8469882

[5] Bloch E.M., Day J.R., Krause P.J., Kjemtrup A., O’Brien S.F., Tobian A.A.R., Goel R. Epidemiology of hospitalized patients with babesiosis, United States, 2010–2016. Emerg. Infect. Dis. 2022; 28:354–362. 35076004 10.3201/eid2802.210213 PMC8798708

[6] Swanson M., Pickrel A., Williamson J., Montgomery S. Trends in reported babesiosis cases—United States, 2011–2019. MMWR Morb. Mortal. Wkly Rep. 2023; 72:273–277. 36928071 10.15585/mmwr.mm7211a1 PMC10027409

[7] Gerace E., Lo Presti V.D.M., Biondo C. *Cryptosporidium* infection: epidemiology, pathogenesis, and differential diagnosis. Eur. J. Microbiol. Immunol. 2019; 9:119–123. 10.1556/1886.2019.00019 PMC6945992 31934363

[8] Liu Q., Wang Z.D., Huang S.Y., Zhu X.Q. Diagnosis of toxoplasmosis and typing of *Toxoplasma gondii*. Parasit. Vectors. 2015; 8:292. 26017718 10.1186/s13071-015-0902-6 PMC4451882

[9] Singh P., Lonardi S., Liang Q., Vydyam P., Khabirova E., Fang T., Gihaz S., Thekkiniath J., Munshi M., Abel S. et al. *Babesia duncani* multi-omics identifies virulence factors and drug targets. Nat. Microbiol. 2023; 8:845–859. 37055610 10.1038/s41564-023-01360-8 PMC10159843

[10] Amos B., Aurrecoechea C., Barba M., Barreto A., Basenko E.Y., Bazant W., Belnap R., Blevins A.S., Bohme U., Brestelli J. et al. VEuPathDB: the eukaryotic pathogen, vector and host bioinformatics resource center. Nucleic Acids Res. 2022; 50:D898–D911. 34718728 10.1093/nar/gkab929 PMC8728164

[11] Giraldo-Calderon G.I., Harb O.S., Kelly S.A., Rund S.S., Roos D.S., McDowell M.A. VectorBase.org updates: bioinformatic resources for invertebrate vectors of human pathogens and related organisms. Curr. Opin. Insect Sci. 2022; 50:100860. 34864248 10.1016/j.cois.2021.11.008 PMC9133010

[12] Nevers Y., Glover N.M., Dessimoz C., Lecompte O. Protein length distribution is remarkably uniform across the tree of life. Genome Biol. 2023; 24:135. 37291671 10.1186/s13059-023-02973-2 PMC10251718

[13] Tiessen A., Perez-Rodriguez P., Delaye-Arredondo L.J. Mathematical modeling and comparison of protein size distribution in different plant, animal, fungal and microbial species reveals a negative correlation between protein size and protein number, thus providing insight into the evolution of proteomes. BMC Res. Notes. 2012; 5:85. 22296664 10.1186/1756-0500-5-85 PMC3296660

[14] Dragicevic M.B., Paunovic D.M., Bogdanovic M.D., Todorovic S.I., Simonovic A.D. ragp: pipeline for mining of plant hydroxyproline-rich glycoproteins with implementation in R. Glycobiology. 2019; 30:19–35. 10.1093/glycob/cwz072 31508799

[15] Li L., Stoeckert C.J. Jr, Roos D.S OrthoMCL: identification of ortholog groups for eukaryotic genomes. Genome Res. 2003; 13:2178–2189. 12952885 10.1101/gr.1224503 PMC403725

[16] Buchfink B., Reuter K., Drost H.G. Sensitive protein alignments at tree-of-life scale using DIAMOND. Nat. Methods. 2021; 18:366–368. 33828273 10.1038/s41592-021-01101-x PMC8026399

[17] Que X., Ngo H., Lawton J., Gray M., Liu Q., Engel J., Brinen L., Ghosh P., Joiner K.A., Reed S.L. The cathepsin B of *Toxoplasma gondii*, toxopain-1, is critical for parasite invasion and rhoptry protein processing. J. Biol. Chem. 2002; 277:25791–25797. 12000756 10.1074/jbc.M202659200

[18] Morrissette N., Abbaali I., Ramakrishnan C., Hehl A.B. The tubulin superfamily in apicomplexan parasites. Microorganisms. 2023; 11:706. 36985278 10.3390/microorganisms11030706 PMC10056924

[19] Carruthers V.B., Tomley F.M. Microneme proteins in apicomplexans. Subcell. Biochem. 2008; 47:33–45. 18512339 10.1007/978-0-387-78267-6_2 PMC2847500

[20] Sloan M.A., Aghabi D., Harding C.R. Orchestrating a heist: uptake and storage of metals by apicomplexan parasites. Microbiology (Reading). 2021; 167:mic.0.001114. 34898419 10.1099/mic.0.001114 PMC7612242

[21] Alexandrov K., Horiuchi H., Steele-Mortimer O., Seabra M.C., Zerial M. Rab escort protein-1 is a multifunctional protein that accompanies newly prenylated rab proteins to their target membranes. EMBO J. 1994; 13:5262–5273. 7957092 10.1002/j.1460-2075.1994.tb06860.x PMC395482

[22] Kolakowski D., Rzepnikowska W., Kaniak-Golik A., Zoladek T., Kaminska J. The GTPase Arf1 is a determinant of yeast Vps13 localization to the Golgi apparatus. Int. J. Mol. Sci. 2021; 22:12274. 34830155 10.3390/ijms222212274 PMC8619211

[23] Ruggeri Z.M., Ware J. von Willebrand factor. FASEB J. 1993; 7:308–316. 8440408 10.1096/fasebj.7.2.8440408

[24] Neuwald A.F., Aravind L., Spouge J.L., Koonin E.V. AAA+: a class of chaperone-like ATPases associated with the assembly, operation, and disassembly of protein complexes. Genome Res. 1999; 9:27–43. 9927482

[25] Chen Y., Kelly E.E., Masluk R.P., Nelson C.L., Cantu D.C., Reilly P.J. Structural classification and properties of ketoacyl synthases. Protein Sci. 2011; 20:1659–1667. 21830247 10.1002/pro.712 PMC3218358

[26] Jackson D.R., Tu S.S., Nguyen M., Barajas J.F., Schaub A.J., Krug D., Pistorius D., Luo R., Muller R., Tsai S.C. Structural insights into anthranilate priming during type II polyketide biosynthesis. ACS Chem. Biol. 2016; 11:95–103. 26473393 10.1021/acschembio.5b00500 PMC7886370

[27] Lampert I.A., Jones P.D., Sadler T.E., Castro J.E. Intravascular coagulation resulting from intravenous injection of *C. parvum* in mice. Br. J. Cancer. 1977; 36:15–22. 889682 10.1038/bjc.1977.149 PMC2025445

[28] Rank G., Sutton R., Marshall V., Lundie R.J., Caddy J., Romeo T., Fernandez K., McCormack M.P., Cooke B.M., Foote S.J. et al. Novel roles for erythroid ankyrin-1 revealed through an ENU-induced null mouse mutant. Blood. 2009; 113:3352–3362. 19179303 10.1182/blood-2008-08-172841 PMC2665900

[29] Wrzesinski J., Nurse K., Bakin A., Lane B.G., Ofengand J. A dual-specificity pseudouridine synthase: an *Escherichia coli* synthase purified and cloned on the basis of its specificity for ψ746 in 23S RNA is also specific for ψ32 in tRNA^phe^. RNA. 1995; 1:437–448. 7493321 PMC1482406

[30] Adam R.D., Nigam A., Seshadri V., Martens C.A., Farneth G.A., Morrison H.G., Nash T.E., Porcella S.F., Patel R. The *Giardia lamblia* vsp gene repertoire: characteristics, genomic organization, and evolution. BMC Genomics. 2010; 11:424. 20618957 10.1186/1471-2164-11-424 PMC2996952

[31] Cardone G., Newcomb W.W., Cheng N., Wingfield P.T., Trus B.L., Brown J.C., Steven A.C. The UL36 tegument protein of herpes simplex virus 1 has a composite binding site at the capsid vertices. J. Virol. 2012; 86:4058–4064. 22345483 10.1128/JVI.00012-12 PMC3318633

[32] Blaho J.A., Roizman B. ICP4, the major regulatory protein of herpes simplex virus, shares features common to GTP-binding proteins and is adenylated and guanylated. J. Virol. 1991; 65:3759–3769. 1645791 10.1128/jvi.65.7.3759-3769.1991 PMC241406

[33] Hviid L., Jensen A.T. PfEMP1—a parasite protein family of key importance in *Plasmodium falciparum* malaria immunity and pathogenesis. Adv. Parasitol. 2015; 88:51–84. 25911365 10.1016/bs.apar.2015.02.004

[34] Cubillos E.F.G., Snebergerova P., Borsodi S., Reichensdorferova D., Levytska V., Asada M., Sojka D., Jalovecka M. Establishment of a stable transfection and gene targeting system in *Babesia divergens*. Front. Cell. Infect. Microbiol. 2023; 13:1278041. 38156314 10.3389/fcimb.2023.1278041 PMC10753763

[35] Jaijyan D.K., Govindasamy K., Singh J., Bhattacharya S., Singh A.P. Establishment of a stable transfection method in *Babesia microti* and identification of a novel bidirectional promoter of *Babesia microti*. Sci. Rep. 2020; 10:15614. 32973208 10.1038/s41598-020-72489-3 PMC7515924

[36] Liu M., Ji S., Rizk M.A., Adjou Moumouni P.F., Galon E.M., Li J., Li Y., Zheng W., Benedicto B., Tumwebaze M.A. et al. Transient transfection of the zoonotic parasite *Babesia microti*. Pathogens. 2020; 9:108. 32050586 10.3390/pathogens9020108 PMC7169379

[37] Wang S., Li D., Chen F., Jiang W., Luo W., Zhu G., Zhao J., He L. Establishment of a transient and stable transfection system for *Babesia duncani* using a homologous recombination strategy. Front. Cell. Infect. Microbiol. 2022; 12:844498. 35463640 10.3389/fcimb.2022.844498 PMC9019647

[38] Giorgalli M., Cunningham D.A., Broncel M., Sait A., Harrison T.E., Hosking C., Vandomme A., Amis S.I., Antonello A., Sullivan L. et al. Differential trafficking and expression of PIR proteins in acute and chronic *Plasmodium* infections. Front. Cell. Infect. Microbiol. 2022; 12:877253. 35782145 10.3389/fcimb.2022.877253 PMC9245118

[39] Verweij J.J., Blange R.A., Templeton K., Schinkel J., Brienen E.A., van Rooyen M.A., van Lieshout L., Polderman A.M. Simultaneous detection of *Entamoeba histolytica*, *Giardia lamblia*, and *Cryptosporidium parvum* in fecal samples by using multiplex real-time PCR. J. Clin. Microbiol. 2004; 42:1220–1223. 15004079 10.1128/JCM.42.3.1220-1223.2004 PMC356880

[40] Martinez C., Marzec T., Smith C.D., Tell L.A., Sehgal R.N. Identification and expression of maebl, an erythrocyte-binding gene, in *Plasmodium gallinaceum*. Parasitol. Res. 2013; 112:945–954. 23224610 10.1007/s00436-012-3211-4 PMC3581715

[41] Alzan H.F., Knowles D.P., Suarez C.E. Comparative bioinformatics analysis of transcription factor genes indicates conservation of key regulatory domains among *Babesia bovis*, *Babesia microti*, and *Theileria equi*. PLoS Negl. Trop. Dis. 2016; 10:e0004983. 27832060 10.1371/journal.pntd.0004983 PMC5104403

[42] Gebreegziabher Amare M., Westrick N.M., Keller N.P., Kabbage M. The conservation of IAP-like proteins in fungi, and their potential role in fungal programmed cell death. Fungal Genet. Biol. 2022; 162:103730. 35998750 10.1016/j.fgb.2022.103730

[43] Cai X., Herschap D., Zhu G. Functional characterization of an evolutionarily distinct phosphopantetheinyl transferase in the apicomplexan *Cryptosporidium parvum*. Eukaryot. Cell. 2005; 4:1211–1220. 16002647 10.1128/EC.4.7.1211-1220.2005 PMC1168963

[44] Mazumdar J., Striepen B. Make it or take it: fatty acid metabolism of apicomplexan parasites. Eukaryot. Cell. 2007; 6:1727–1735. 17715365 10.1128/EC.00255-07 PMC2043401

[45] Cornillot E., Hadj-Kaddour K., Dassouli A., Noel B., Ranwez V., Vacherie B., Augagneur Y., Bres V., Duclos A., Randazzo S. et al. Sequencing of the smallest apicomplexan genome from the human pathogen *Babesia microti*. Nucleic Acids Res. 2012; 40:9102–9114. 22833609 10.1093/nar/gks700 PMC3467087

[46] Cuesta I., Gonzalez L.M., Estrada K., Grande R., Zaballos A., Lobo C.A., Barrera J., Sanchez-Flores A., Montero E. High-quality draft genome sequence of *Babesia divergens*, the etiological agent of cattle and human babesiosis. Genome Announc. 2014; 2:e01194-14. 25395649 10.1128/genomeA.01194-14 PMC4241675

[47] Jackson A.P., Otto T.D., Darby A., Ramaprasad A., Xia D., Echaide I.E., Farber M., Gahlot S., Gamble J., Gupta D. et al. The evolutionary dynamics of variant antigen genes in *Babesia* reveal a history of genomic innovation underlying host–parasite interaction. Nucleic Acids Res. 2014; 42:7113–7131. 24799432 10.1093/nar/gku322 PMC4066756

[48] Kremer K., Kamin D., Rittweger E., Wilkes J., Flammer H., Mahler S., Heng J., Tonkin C.J., Langsley G., Hell S.W. et al. An overexpression screen of *Toxoplasma gondii* Rab-GTPases reveals distinct transport routes to the micronemes. PLoS Pathog. 2013; 9:e1003213. 23505371 10.1371/journal.ppat.1003213 PMC3591302

