# Supplementary material for: Properties and predicted functions of large genes and proteins of apicomplexan parasites
Source: NAR Genom Bioinform. 2024 Apr 4;6(2):lqae032. doi: 10.1093/nargab/lqae032 (PMC10993292; doi:10.1093/nargab/lqae032)
Supplement: lqae032_Supplemental_Files [file lqae032_supplemental_files.zip › Supplemental Figures legends.docx]

**Supplemental Figures**

**Figure S1.** Barplots showing the Pearson correlation coefficients of protein length in each species to the fractional composition per amino acid. Negative correlations mean that abundance of a certain amino acid typically corresponds with shorter proteins.

**Figure S2.** Barplots showing the Pearson correlation coefficients of protein length in the large proteins of each species to the fractional composition per amino acid. Negative correlations mean that abundance of a certain amino acid typically corresponds with shorter proteins.

**Figure S3.** Heatmap representing codon usage across all parasitic genomes and *S. cerevisiae* and *E. histolytica* outgroups measured in frequency per thousand codons.

**Figure S4.** Scatterplots with trendlines depicting amino acid content over protein size for: (top left) cysteine in the whole *B. duncani* proteome, (top right) cysteine in *B. duncani* large proteins, (bottom left) asparagine in the whole *P. falciparum* proteome, (bottom right) asparagine in *P. falciparum* large proteins.
