# Supplementary material for: Properties and predicted functions of large genes and proteins of apicomplexan parasites
Source: NAR Genom Bioinform. 2024 Apr 4;6(2):lqae032. doi: 10.1093/nargab/lqae032 (PMC10993292; doi:10.1093/nargab/lqae032)
Supplement: lqae032_Supplemental_Files [file lqae032_supplemental_files.zip › Table S1.pdf]

Table S1. Functional Profiling of Conserved Domains of Largest Proteins in Apicomplexa

Species	Gene ID	Protein length (aa)	TM domains	CD Descriptions	Transport + Signaling	Metabolism	DNA/RNA/ Protein synthesis
B. divergens	Bdiv_001880c	5030	0	VPS13			
				MRS6			
B. duncani	BdWA1_000001	11561	18	No conserved domains found			
B. microti	BmR1_04g05531	4337	0	MDN1 (midasin)			
				vWFA			
C. parvum	cgd4_2900	13413	0	Polyketide synthase			
				AFD Class I			
				NAD Binding 4			
				Kringle domain			
				NADB Rossmann			
				Hot Dog			
				Phospho-pantethine binding			
				MDR			
				Keto-acyl synthetase			
P. falciparum	PF3D7_0628100	10287	2	HECT domain			
				Ankyrin 2			
P. vivax	PVP01_1022500	11461	0	Pseudouridylate synthase			
				PTZ00121 (malarial adhesin-like protein)			
				MATH domain			
				PTZ00449 (microneme/rhoptry)			
				2A1904			
T. gondii	TGME49_280660	17226	0	HECT domain			
