# Supplementary material for: Properties and predicted functions of large genes and proteins of apicomplexan parasites
Source: NAR Genom Bioinform. 2024 Apr 4;6(2):lqae032. doi: 10.1093/nargab/lqae032 (PMC10993292; doi:10.1093/nargab/lqae032)
Supplement: lqae032_Supplemental_Files [file lqae032_supplemental_files.zip › Table S2.pdf]

Table S2. Functional Profiling of Pfam Domains of Largest Proteins in Apicomplexa

Species	Gene ID	Protein length (aa)	Ortholog Group	Associated Pfam Domains	Transport + Signaling	Metabolism	DNA/RNA/ Protein synthesis
B. divergens	Bdiv_001880c	5030	OG6_194529	VPS13			
				Chorein-N			
B. duncani	BdWA1_000001	11561	No Pfam domains found				
B. microti	BmR1_04g05531	4337	OG6_100908	AAA domain (dynein-related subfamily) x6			
C. parvum	cgd4_2900	13413	OG6_100178	Acyl transferase x2			
				Alcohol dehydrogenase GroES-like			
				AMP-binding enzyme			
				Beta-ketoacyl synthase x14			
				KR x4			
				Male sterility protein			
				Phosphopantethine attachment x8			
				Polyketide synthase dehydratase x3			
				Zinc-binding dehydrogenase x3			
P. falciparum	PF3D7_0628100	10287	OG6_126864	Ankyrin repeats			
P. vivax	PVP01_1022500	11461	OG6_533345	Pseudouridylate synthase			
				PTZ00121 (malarial adhesin-like protein)			
				MATH domain			
				PTZ00449 (microneme/rhoptry)			
				2A1904			
T. gondii	TGME49_280660	17226	OG6_139467	HECT domain			
