# Supplementary material for: Properties and predicted functions of large genes and proteins of apicomplexan parasites
Source: NAR Genom Bioinform. 2024 Apr 4;6(2):lqae032. doi: 10.1093/nargab/lqae032 (PMC10993292; doi:10.1093/nargab/lqae032)
Supplement: lqae032_Supplemental_Files [file lqae032_supplemental_files.zip › Table S3.pdf]

Table S3. Definitions of Significantly (p<0.001) overrepresented GO ID’s in large proteins (reference for Fig. 5)

GO Term ID	Definition
GO:0003677	DNA binding
GO:0005388	P-type calcium transporter activity
GO:0016787	hydrolase activity
GO:0046872	metal ion binding
GO:0000166	nucleotide binding
GO:0005524	ATP binding
GO:0003777	microtubule motor activity
GO:0005515	protein binding
GO:0004114	3',5'-cyclic-nucleotide phosphodiesterase activity
GO:0008081	phosphoric diester hydrolase activity
GO:0003676	nucleic acid binding
GO:0004687	myosin light chain kinase activity
GO:0004672	protein kinase activity
GO:0003700	DNA-binding transcription factor activity
GO:0016887	ATP hydrolysis activity
GO:0004386	helicase activity
GO:0005096	GTPase activator activity
GO:0008061	chitin binding
GO:0140326	ATPase-coupled intramembrane lipid transporter activity
GO:0003887	DNA-directed DNA polymerase activity
GO:0003774	cytoskeletal motor activity
GO:0016740	transferase activity
GO:0003723	RNA binding
GO:0003824	catalytic activity
GO:0016779	nucleotidyltransferase activity
GO:0005085	guanyl-nucleotide exchange factor activity
GO:0003779	actin binding
GO:0051015	actin filament binding
GO:0003843	1,3-beta-D-glucan synthase activity
GO:0070615	N/A
GO:0003678	xenobiotic transport
GO:0004523	RNA-DNA hybrid ribonuclease activity
GO:0003964	RNA-directed DNA polymerase activity
GO:0004190	aspartic-type endopeptidase activity
GO:0004519	endonuclease activity
GO:0004518	nuclease activity
GO:0008233	peptidase activity
GO:0042626	ATPase-coupled transmembrane transporter activity
GO:0017056	structural constituent of nuclear pore
GO:0004674	protein serine/threonine kinase activity
GO:0008569	minus-end-directed microtubule motor activity
GO:0043015	gamma-tubulin binding
GO:0046789	host cell surface receptor binding
GO:0005525	GTP binding
GO:0003924	GTPase activity
GO:0008559	ABC-type xenobiotic transporter activity
GO:0004527	exonuclease activity
GO:0016874	ligase activity
GO:0016021	N/A
GO:0006260	DNA replication
GO:0003899	DNA-directed 5'-3' RNA polymerase activity
GO:0140359	ABC-type transporter activity
GO:0042908	xenobiotic transport
