# Supplementary figures and images for: Properties and predicted functions of large genes and proteins of apicomplexan parasites
Source: NAR Genom Bioinform. 2024 Apr 4;6(2):lqae032. doi: 10.1093/nargab/lqae032 (PMC10993292; doi:10.1093/nargab/lqae032)

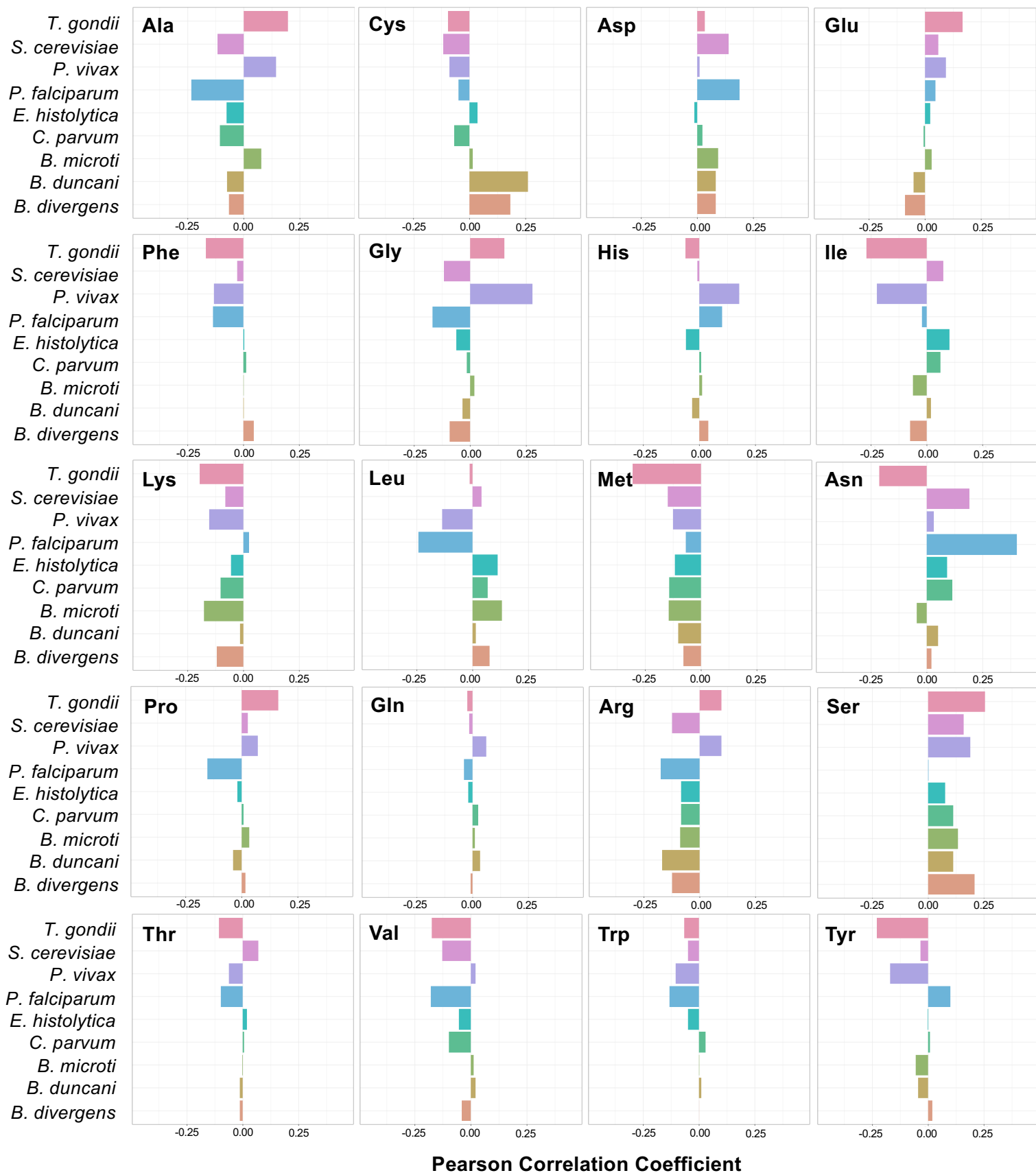

**Figure S1**

Supplement: lqae032_Supplemental_Files [file lqae032_supplemental_files.zip › Fig S1.pdf]

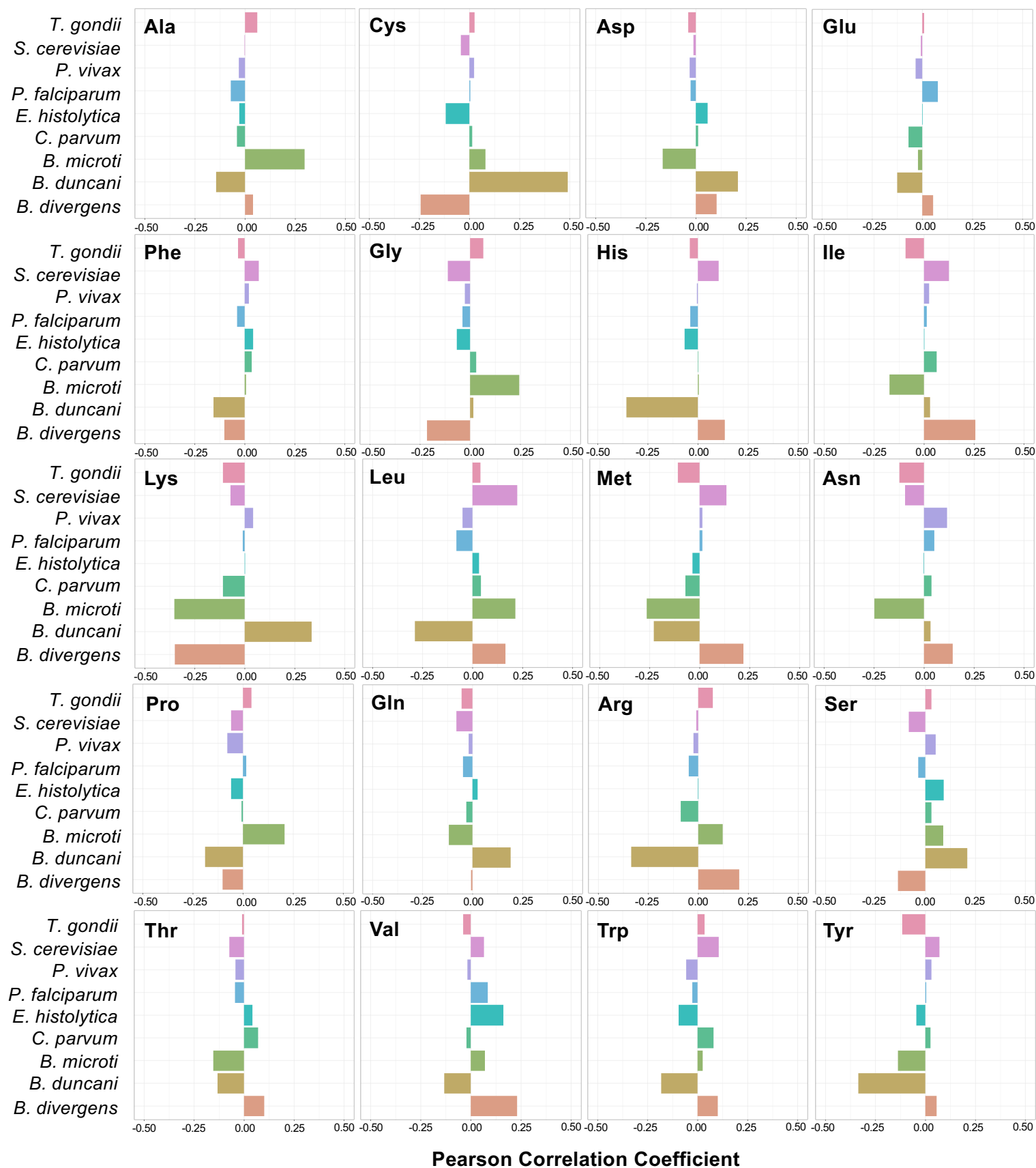

**Figure S2**

Supplement: lqae032_Supplemental_Files [file lqae032_supplemental_files.zip › Fig S2.pdf]

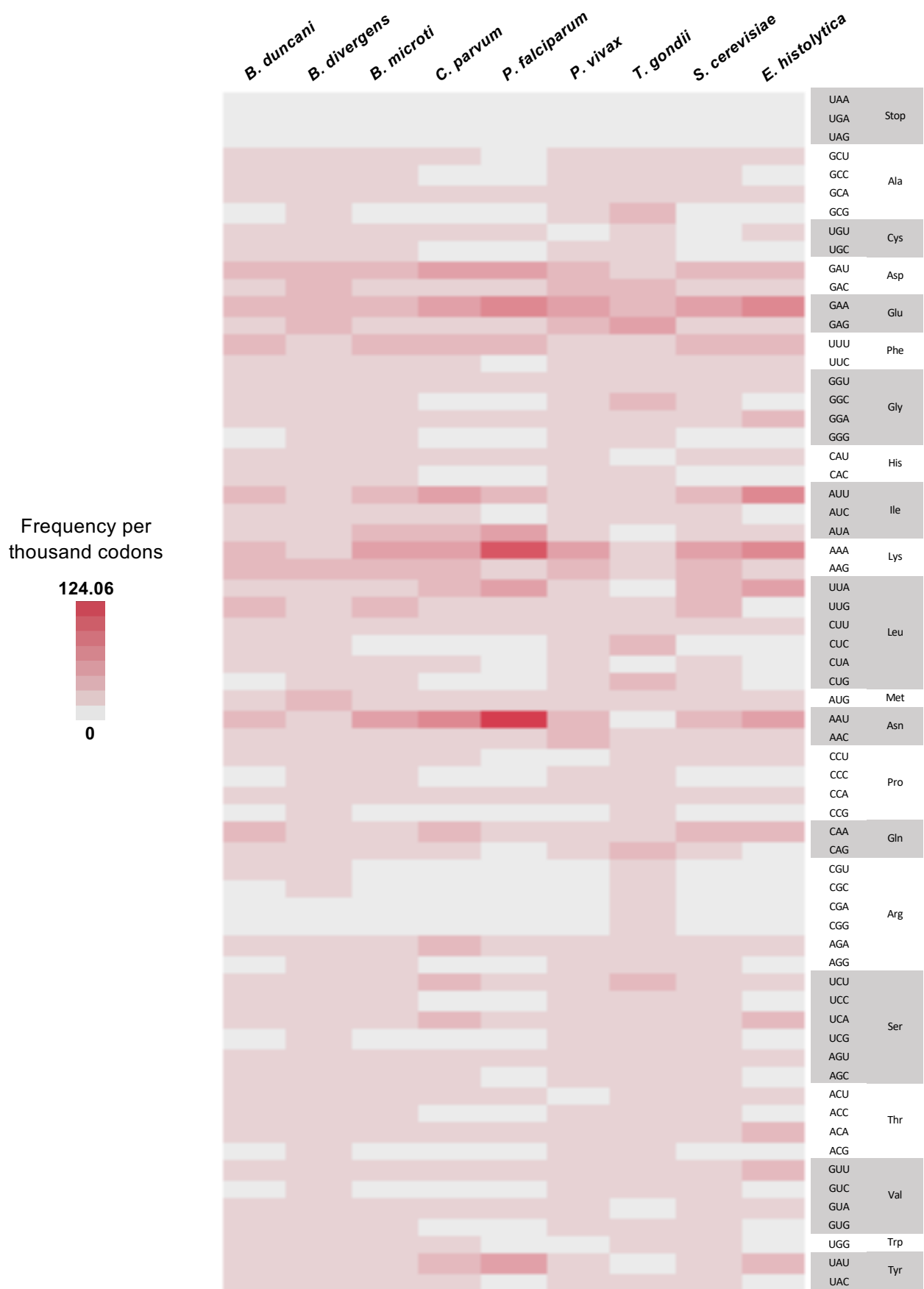

Figure S3

Supplement: lqae032_Supplemental_Files [file lqae032_supplemental_files.zip › Fig S3.pdf]

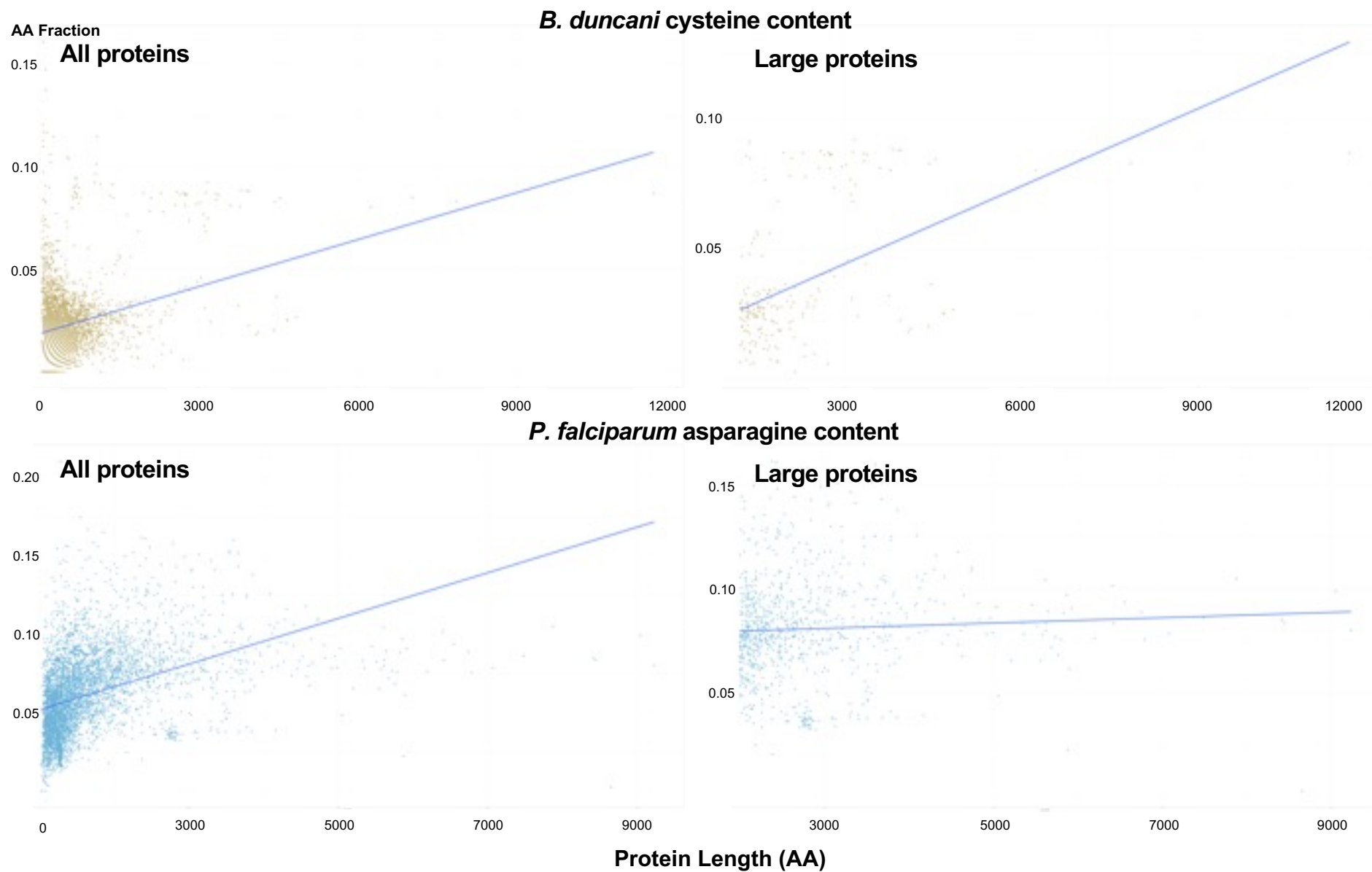

**Fig. S4**

Supplement: lqae032_Supplemental_Files [file lqae032_supplemental_files.zip › Figure S4.pdf]
